# Comparison of the Repeatability and Reproducibility Levels of ANB, Tau and Yen Angle Measurements Used in Cephalometric Diagnostics in the Assessment of Sagittal Discrepancy: A Comparative Study

**DOI:** 10.3390/jcm14072408

**Published:** 2025-04-01

**Authors:** Jacek Kotuła, Konrad Szendoł, Krzysztof Kotuła, Wojciech Dobrzyński, Joanna Lis, Beata Kawala, Michał Sarul, Anna Ewa Kuc

**Affiliations:** 1 Department of Dentofacial Orthopedics and Orthodontics, Wroclaw Medical University, Krakowska 26, 50-425 Wroclaw, Poland; konrad.szendol@gmail.com (K.S.); beata.kawala@umw.edu.pl (B.K.); annaewakuc@wp.pl (A.E.K.); 2 Faculty of Medicine, Pomeranian Medical University, 70-204 Szczecin, Poland; krzyskotula@gmail.com; 3 Division of Facial Abnormalities, Department of Dentofacial Orthopedics and Orthodontics, Wroclaw Medical University, Wroclaw Medical University, Krakowska 26, 50-425 Wroclaw, Poland; wojt.dobrzynski@wp.pl (W.D.); joanna.lis@umed.wroc.pl (J.L.); 4 Department of Integrated Dentistry, Wroclaw Medical University, Krakowska 26, 50-425 Wroclaw, Poland; michal.sarul@umw.edu.pl

**Keywords:** cephalometry, orthodontics, reproducibility of results, sagittal plane

## Abstract

**Introduction**: Cephalometric analysis is an essential tool used in orthodontic diagnosis and treatment planning. **Aim**: The aim of this study was to compare the measurement reliabilities (repeatability and reproducibility) of the Tau and Yen angles and compare them to the results obtained for the ANB angle. **Methods**: Repeatability and reliability assessments for the seven points (N, A, B, S, W, M, G) used in the analysis of ANB, Yen and Tau angles were performed twice with an interval of 7 days by 22 orthodontists. The measurement results for ANB, Yen and Tau angles were assessed using the Bland–Altman formula, Dahlberg formula, intraclass correlation coefficients (ICCs), R^2^ coefficients and R&R. In order to assess the number of individual skeletal classes of sagittal discrepancy, the Pearson chi-squared test was used. With common parameters of df = 4, *p* < 0001, for the ANB angle, the result was χ^2^ = 9104; for the Tau angle, χ^2^ = 4556; and for the Yen angle, χ^2^ = 4207. In order to determine the inter-rater reliability based on two-way ANOVA analysis without repetitions, the ICC (2,2) was used. The ICC (2,2) index at the 95% confidence level was 0.998 for the ANB angle, 0.997 for Tau and 0.998 for Yen. High values of the ICC index close to 1 indicate the agreement of the measurements and their high reliability. **Results**: The orthodontists in the study measured sagittal discrepancy significantly more accurately using the ANB angle compared to the Yen and Tau angles. Using a Bland–Altman plot, the bias and range of agreement within which 95% of the differences between measurements were accounted for were determined. For the ANB angle, the mean difference between measurements was 0.07 with a confidence interval of −1.55 to +1.69; for the Tau angle, the mean difference between measurements was 0.19 with a confidence interval of −2.92 to 3.30; and for the Yen angle, the mean difference was 0.09 with a confidence interval of −2.71 to +2.89. Using regression analysis, the measurements were assessed using the R2 index, which for the ANB angle was 0.952 (*p* < 0.001); for the Tau angle, R2 = 0.928 (*p* < 0.001), and for the Yen angle, R2 = 0.942 (*p* < 0.001). **Conclusions:** The obtained results of the assessment of the ANB, Tau and Yen angles confirm the thesis of the highest reliability, including repeatability and reproducibility, in the assessment of sagittal discrepancy in orthodontic diagnostics using the ANB angle, previously considered the gold standard. One of the basic factors attributed to the poorer repeatability and reproducibility of Tau and Yen measurements is human error related to the precision of determining new anthropometric points. Further studies to assess the usefulness of using the new Tau and Yen angle measurements in orthodontic diagnostics for sagittal discrepancy should be correlated with other measurements used so far, depending on the type of defects in the vertical dimension. It is necessary to consider enlarging the study group and performing longitudinal studies.

## 1. Introduction

The analysis of lateral cephalograms is a key element in orthodontic diagnostics and treatment planning [1,2]. In order to perform correct diagnostics using cephalometric analysis, it is necessary to maintain the reliability of determining anthropometric points [3,4,5,6,7]. This allows for the obtainment of reliable (repeatable and reproducible) angular measurements assessing the sagittal discrepancy of the maxillary bases [8,9,10,11,12,13]. The precision of determining reference points, despite the dynamic development of medical knowledge and imaging techniques, is subject to multifactorial limitations [14,15]. Theoretically, these can be overcome by using newly developed modern technologies, specifically artificial intelligence (AI), in the technical stages of introducing reference points and performing cephalometric measurements, but there has been a long-standing discussion in the literature on the accuracy and repeatability of measurements performed by operators compared to those generated by AI [16,17,18,19,20], and this dispute remains unresolved after a systematic review [20]. Previous studies on the repeatability and reliability of the diagnosis of skeletal sagittal incongruence were mainly based on reference points used to determine the ANB angle, the values of which were individualized using the Segner and Hasund method [21]. Other parameters were also assessed, the most prominent of which are Wits and the Beta angle. However, each of these parameters raises certain concerns about the correct assessment of sagittal incongruence. The low stability of the ANB angle is due to the instability of the N point position due to bone layering during growth and the change in the ANB angle size during growth. Changes in the length of the skull base during growth also affect the instability of this parameter. The Wits assessment, related to the instability of the occlusal plane, is also not free from error. It depends on the correct determination of the occlusal plane, which is often difficult at individual stages of development and tooth eruption. The course of the occlusal plane is also affected by missing teeth or mandibular deformities. This reduces the reliability of this measurement. The Beta angle assessment [1,2] was developed to facilitate the possibility of proper assessment of sagittal discrepancy in connection with the limitations of the ANB angle and the Wits measurement. The Beta angle is formed by a perpendicular line drawn from point C to point A, intersecting with the AB line. However, this parameter is also burdened with the risk of error [1,2]. First, similarly to the ANB angle, it is based on point A, which is unstable and changes its position during the growth and reconstruction of the alveolar process during orthodontic treatment. In addition, it depends on the axis of the mandibular condyle, the position of which may be difficult to determine. Both of these difficulties limit the reliability of the Beta angle.

Considering the limitations of the discussed methods, practitioners to this day try to combine individual parameters such as the ANB angle and the Wits assessment in order to properly assess the sagittal discrepancy of the maxillary bases.

The current limitations affect the systematic search for new, more reliable methods for assessing the sagittal discrepancy of the maxillary bases. A systematic review conducted in 2022 by Kotuła et al. [1] revealed new cephalometric measurements in the assessment of sagittal discrepancy. Among those listed were the Yen [22] and Tau [23] angles based on new cephalometric points: M (center of the anterior maxilla), G (center at the bottom of the mandibular symphysis) and T for the Tau angle (located at the highest point at the junction of the frontal wall of the pituitary fossa and the sellar tubercle). Previous studies [2] did not confirm higher effectiveness for the assessment of sagittal discrepancy of the Tau parameter. Therefore, the possibility of their widespread introduction in orthodontic diagnostics requires an objective assessment of reliability, including the reproducibility and repeatability of the new—compared to A, N and B—points G, M and T, excluding the still-controversial AI. This study aims to evaluate the reliability (repeatability and reproducibility) of Tau and Yen angle measurements based on the new G and M points (between the lines connecting TGM and SMG), comparing them to the results obtained for the ANB angle in the diagnosis and treatment planning of sagittal discrepancies.

## 2. Materials and Methods

### 2.1. Materials

The study was approved by the Bioethics Committee of the District Medical Chamber in Zielona Góra (decision 01/173/2023 of 6 March 2023). Informed consent, according to the guidelines of the Declaration of Helsinki [24], was obtained from all participants taking part in the study and/or their guardians. All subjects were selected to meet the inclusion criteria, representing Caucasian patients aged 12–18 years, prior to orthodontic treatment, with an orthognathic face (79° ≤ SNA ≤ 85°) with different angle class I, class II and class III categories characterized by different base angles and fulfilling the conditions of the absence of systemic diseases or untreated dental or periodontal disease. Each patient was underwent lateral cephalogram in habitual occlusion, maintaining the parallelism of the Frankfurt plane to the ground and the perpendicularity of the beam to the sagittal plane. After the visual evaluation of the cephalograms, the following criteria were applied for exclusion from the study:

1. Asymmetry interpreted as a greater than 8 mm divergence of the contours of the right and left sides of the mandibular body;

2. Projection error or incorrect contrast preventing the identification of reference points;

3. Shifts in bilateral anatomical structures relative to each other.

The study selected X-ray images of generally healthy patients, without developmental defects of the facial skeleton or ongoing periodontal disease.

### 2.2. Methods

The sample size was calculated using G*Power software (Kiel University, Germany) based on initial measurements with a significance level of *p* = 0.05, d = 0.5, a confidence interval of 95% and a power of 85% for 89 digital lateral cephalograms, recorded as the last 6 digits of the patients’ PESEL numbers. All cephalograms were analyzed based on reference points entered into the computer in the Ortodoncja V.9 program (Ortobajt^®^, Wroclaw, Poland) using NEC Multisync EA 244 WMI Monitors of laboratory-certified high quality (NEC, Tokyo, Japan). Monitor specifications were as follows: size 24 inches, resolution 1920 X 1200, pixel pitch 0.270 mm, viewing angle 178° vertical and 178° horizontal, contrast 1000:1.

**Figure 1:**
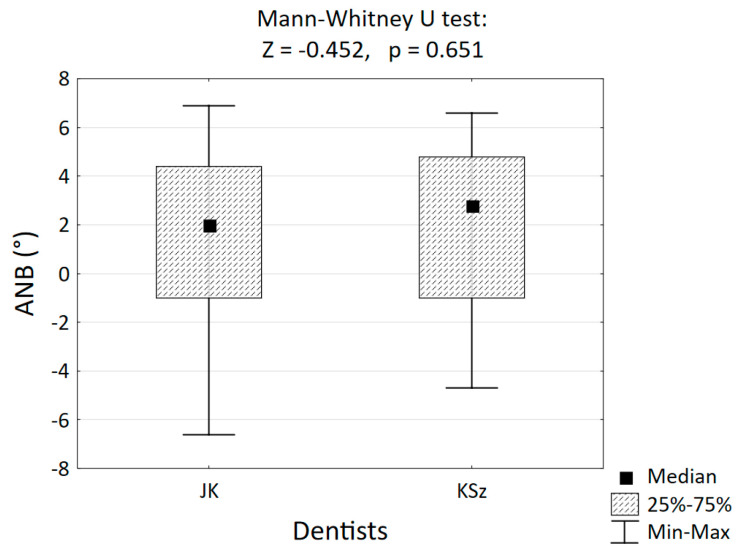
Results of the ANB angle measurements performed by the lead authors (JK and KSz) on 89 cephalograms.

The two lead authors of the article (JK and KSz) independently analyzed the database of consecutive X-ray images using the Segner and Hasund method, measured the ANB angle and randomly selected 89 radiographs for analysis from the database in such a way as to obtain three groups of patients with similar numbers of skeletal classes I, II and III. The agreement of the assessments of both orthodontists when classifying the 89 cephalograms into one of the three classes was checked by ensuring that the value of the Fleiss kappa coefficient of agreement (K) = 0.730 and Cramér’s coefficient V = 0.751. The values of the Fleiss kappa and Cramér’s coefficient indicate good agreement of both orthodontists in classifying patients into skeletal classes. The agreement of the measurement results obtained by both doctors was also confirmed using the Mann–Whitney U test (Figure 1). There was no statistically significant difference between dentists JK and KSz in terms of the ANB angle measurement results (*p* > 0.05).

Then, 89 radiographs were randomly selected for analysis from the database to obtain three groups of patients with similar numbers of skeletal class I, II and III (Table 1).

The corresponding ranges of the individual classes according to the ANB angle measurement were assigned appropriate values for the Yen and Tau angles (Table 2).

Using ROC curve analyses, the ranges of Yen and Tau angle values corresponding to the three skeletal classes determined based on the ANB angle were determined (Table 1). Flaiss’ kappa coefficient of agreement and Cramér’s V coefficient confirmed the agreement of the measurement results obtained by both authors. Then, 89 radiographs were randomly selected for analysis from the database in such a way as to obtain three groups of patients with an orthognathic face (79° ≤ SNA ≤ 85°) with skeletal classes I, II and III according to the ANB angle, to whom the appropriate values of Tau and Yen angles were simultaneously assigned (Table 2). 

The identification of landmarks was performed manually on digital images using a cursor controlled by a computer mouse in the Orthodontics 9.0 program. Each cephalometric image was entered into the program and initially calibrated by KSz using a measuring ruler placed in the calibration window of the program at the level of 3 cm. The images prepared in this way after initial calibration were sent to all participants of the study.

Landmark identification was performed manually on digital images using a mouse-controlled cursor. Results were recorded in an Excel spreadsheet (Microsoft, Seattle, WA, USA). Statistical analysis was then performed. The mean and standard deviation of the Tau and Yen angles for each of the 89 cephalograms related to individual skeletal defects in the sagittal plane were measured in correlation with the size of the base angles to assess the stability of the Tau and Yen angles in assessing skeletal defects. The obtained values were then correlated with the parameters from the ANB angle analysis.

In order to blind the study, after making a copy of the folder with the analyzed cephalograms, patient names were randomly replaced with numbers from 1 to 89 and then sent to 22 researchers—orthodontic residents trained in marking not only points A, N and B but also those used to measure the Yen and Tau angles (Figure 1), because the analysis of the latter two was absent in the scientific literature until the end of 2009 [22] and 2020 [23], respectively. Orthodontists were trained in cephalometric analysis during a 5 h theoretical and practical training course concluding with a skills test. The researchers assessed each cephalogram twice, with a seven-day interval between the analyses, under the same environmental conditions in rooms with reduced light levels, obtaining 3916 measurements of each variable for a total of 11,749 measurements. The individual photos were randomly assigned numbers from 1 to 89. The second column consisted of PESEL numbers corresponding to the individual photos and assigned patient numbers, verifying each patient. Before being sent to the orthodontists by the lead authors, the PESEL numbers were blinded so that it was not possible to identify the patient. After completing the double measurements of each cephalogram, the orthodontics program generated a collective file for each researcher, which was sent to the lead authors (JK, KSz). Data obtained by the investigators were placed separately in 44 Excel spreadsheets (Microsoft, Seattle, WA, USA), assigning them—after unblinding—to the patients’ PESEL numbers. The number of each row corresponded exactly to the patient number assigned before sending the photos to the orthodontists by the author JK. This affected the integrity of the results. The values of ANB, Yen and Tau angles measured during each examination were collected in one research spreadsheet.

**Figure 2:**
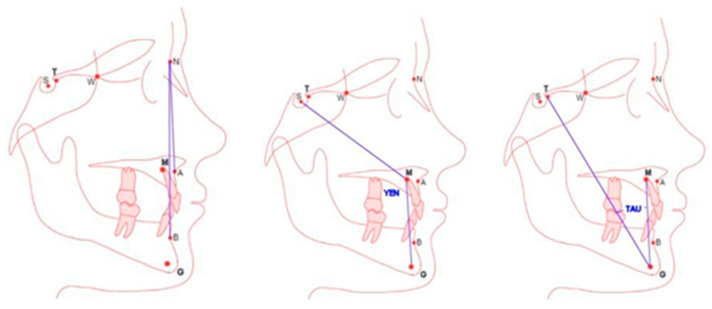
Landmarks A, N, B, T, M and G used in the analysis of ANB, Yen and Tau angles. Location of new cephalometric points T, M and G.

Figure 2 discusses the principles of determining anthropometric points defining angular measurements ANB, Tau and Yen assessing the sagittal discrepancy of maxillary bases in cephalometric diagnostics. The Yen angle is measured at point M between arms SM and MG. The Tau angle is measured at point G between arms TG and GM. The locations of points A, N and B were established according to the method of Segner and Hasund. The locations of new anthropometric points were defined as follows: point T was the highest point at the junction of the anterior wall of the pituitary fossa and the sellar tubercle; point M was the construction point representing the center of the largest circle, tangent to the frontal, upper and palatine surfaces of the maxilla; and point G was the focus of the circle tangent to the inner, frontal, posterior and lower edges of the mandibular-bifid symphysis.

## 3. Statistical Analysis

The analysis, for which the statistics module V.13.3 (Tibco Software Inc., Palo Alto, CA, USA) was used, included the following:

1. Differences in results obtained by the same investigator (repeatability);

2. Differences in results obtained by 22 investigators (reproducibility), influencing the diagnosis of the jaw position in the sagittal dimension (reliability).

For this purpose, the following statistical tools were used: chi-squared test, Bland–Altman plot, Dahlberg formula, R2 coefficient of determination and ICC, the values of which were interpreted according to the standard given by Koo and Li24 and Cohen’s Kappa coefficient with Fleiss correction25.

Dahlberg’s formula was used to estimate the random error between repeated measurements of the same feature.

The next statistical tool used to assess the agreement between two series of measurements was Bland–Altman plots. These show whether the differences between measurement series change depending on the size of the measured values. They also allow for the calculation of mean differences and intervals of agreement.

The strength of the relationship between the results of the first and second measurements of the same parameter under the same conditions was assessed by subtracting the coefficient of determination R2.

The value of the intraclass correlation coefficient (ICC) was used to assess the consistency of the ANB angle measurement results between 22 doctors and 89 patients.

Since each cephalogram was assessed by each of the 22 orthodontists and the agreement between individual measurements was assessed, the ICC model (3,1) was adopted as a measure.

The R&R (repeatability and reproducibility) module of STATISTICA (TIBCO Software Inc., Palo Alto, CA, USA) was also used. The repeatability and reproducibility of the results of the double measurements of the ANB angle in 89 cephalograms performed by 22 orthodontists were assessed. The repeatability of the measurements refers to the comparison between measurements performed by the same orthodontist, while the reproducibility refers to the comparison of measurements performed by different doctors.

According to the evaluation of the index (R&R), the main source of systematic error is the individual variability between patients, which is a positive result, and the assessment skills represented by each orthodontist. In order to minimize the error related to the diversity of patients, patients with an orthognathic face were selected for the study in individual classes of malocclusion I, II and III. In order to minimize human error, training on determining cephalometric points was conducted.

## 4. Results

The selection of 89 cephalometric images from patients with an orthognathic face (79° ≤ SNA ≤ 85°) with skeletal classes I, II and III was performed by the two study leaders. The values of the kappa-Fleiss coefficient (K = 0.730) and Cramér’s coefficient (V = 0.751) obtained to assess the agreement of both leaders indicated good agreement between both orthodontists.

In the group of 22 orthodontists, the classification of cephalometric images sent by the lead authors into one of the three skeletal classes based on the average result of the double ANB angle measurement was highly consistent with the gold standard. The Cohen’s kappa coefficient of agreement was κ = 0.846 and was greater than 0.8, which indicates very good agreement, i.e., high consistency of assessments.

The following statistical tools were used to assess the repeatability of determining the angular measurements ANB, Tau and Yen in assessing sagittal discrepancies:

1. Bland–Altman analysis is a nonparametric method used to assess the agreement between two measurements. This analysis is commonly used to compare a new method of measurement with another or a reference standard. The method involves plotting the differences between two measurements on the *y*-axis against their corresponding values on the *x*-axis. A Bland–Altman plot shows the difference between two measurements as a function of the mean value. Points above the horizontal line (MEAN) indicate that the second measurement tends to be higher than the first, while points below the line indicate that the second measurement tends to be lower than the first.

A Bland–Altman plot is a useful tool for visually and quantitatively assessing the agreement between two measurements, helping to identify both systematic and random errors.

**Figure 3:**
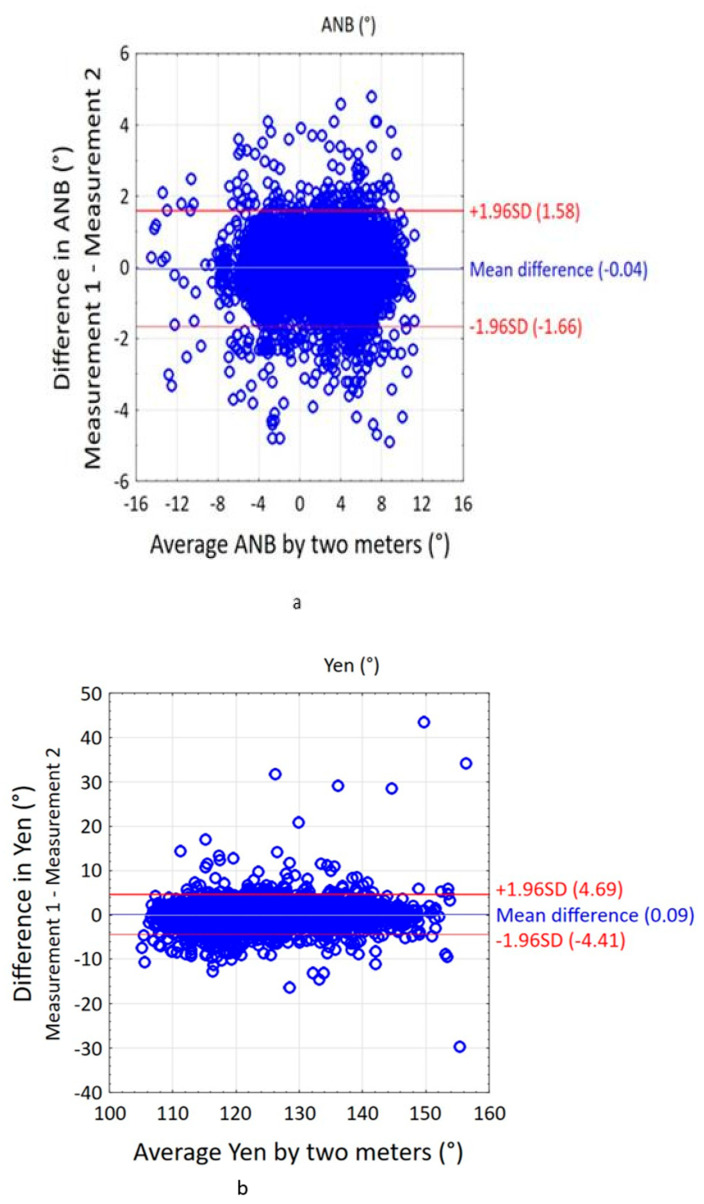

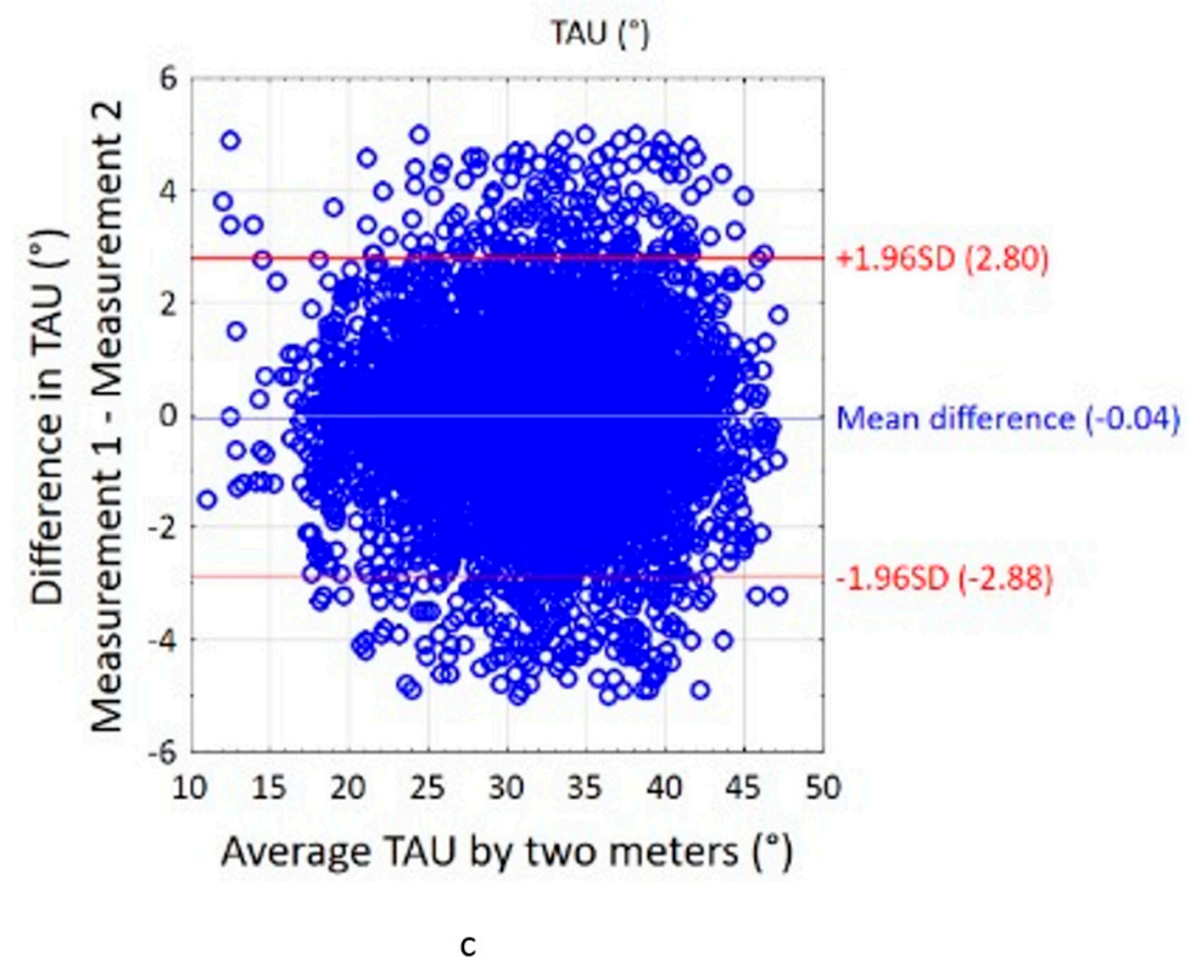
Bland–Altman plot of the two-time measurement of the (**a**) ANB, (**b**) Yen, (**c**) Tau angle.

Bland–Altman plots do not tell us whether the agreement is sufficient to replace one method with another. It simply quantifies the systematic error and the range of agreement within which 95% of the differences between one measurement and the other are considered. We can say that the deviation is small because the line of equality falls within the confidence interval of the mean difference (Figure 3), but only analytical, biological, or clinical considerations can determine whether the interval of agreement is too wide or sufficiently narrow for our purposes. The best way to use the Bland–Altman chart system would be to define a priori the limits of maximum acceptable differences (expected limits of agreement), based on criteria that are biologically and analytically relevant and then calculate statistics to check whether these limits are exceeded or not.

2. The Dahlberg formula allows for the quantitative determination of measurement error made by the same examiner. It allows for the determination of the extent to which the same observer obtains similar results for the same angle at different times. Low error values indicate high repeatability and precision of the observer. A significantly smaller measurement error of one of the angular measurements used in cephalometric analyses to assess sagittal discrepancy indicates higher precision for this parameter. The lower the ME value, the better the repeatability of the angle measurement.

3. Repeatability component of the R&R index: R&R is a statistical indicator relating to the consistency of measurements, both repeatability and reproducibility, and determining the causes of measurement differences. This indicator is calculated as the standard deviation of measurement results and their variance. The following values should be used when interpreting the indicator:

R&R < 10% indicates very good measurement reliability.

10% < R&R < 30% indicates an acceptable level of reliability.

R&R > −30% is characterized by high measurement variability and low reliability.

In the assessment of the Bland–Altman chart, double cephalometric analyses were performed at a time interval of 7 days by the same orthodontist with a 95% confidence level.

### 4.1. Repeatability Analysis

Repeatability refers to the agreement of measurements obtained under the same conditions, by the same examiner, in a short period of time.

Based on the Bland–Altman plot (Figure 3), the results of the repeatability assessment show that it decreased in the following order: ANB, Tau and Yen. The agreement between two series of measurements expresses the average difference between the first and second measurement.

Average differences—close to 0, between the two values of the measured angles ranging from 0.03° for the ANB angle to 0.11° for the Tau angle and −0.10° for the Yen angle—indicate good agreement of the measurements. For the ANB angle, the order of measurements is not observed to have a significant effect on the results. The first measurement does not systematically give higher or lower results compared to the second. The differences between subsequent measurements are randomly distributed and within the limits of agreement, which means that the results of both series are largely consistent. However, a wide spread of the limits of agreement at the level of 95% between the measurements is only acceptable for the ANB angle and amounts to (−1.84–1.91). For the two angles Tau (−6.21–6.43) and Yen (−8.67–8.46), unacceptable limits are characterized by low repeatability of their determination.

$$SE=\sqrt{\frac{\sum {\left(d_{i}\right)}^{2}}{2n}}$$

**Dahlberg’s formula** was used to estimate the random error between repeated measurements of the same feature:

where

SE—standard error of repeated measurements;

di—difference between the first and second measurement of the same feature for the i-th object;

*n*—number of pairs of repeated measurements.

The standard error of repeated measurements for the ANB angle was as follows

$$SE=\sqrt{\frac{\sum {\left(d_{i}\right)}^{2}}{2n}}=0.68$$

**Figure 4:**
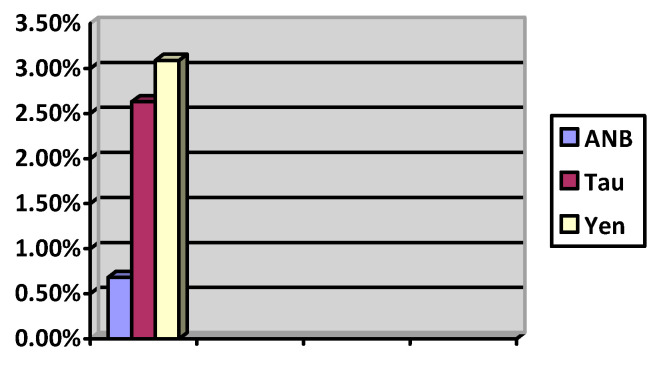
Dahlberg formula of the two-time measurement of the ANB, Yen and Tau angles.

The interpretation of the results obtained with calculations in accordance with the Dahlberg formula allows us to determine that the best repeatability and precision in connection with the lowest ME value (close to 0) are provided by the ANB angle (0.68%). The Tau and Yen values (2.63%, 3.09%) do not allow for comparable precision control in their determination (Figure 4).

### 4.2. Repeatability and Reproducibility Analisis Using the R&R Module

The R&R (repeatability and reproducibility) module of STATISTICA (TIBCO Software Inc., Palo Alto, CA, USA) was also used to assess the repeatability of angular measurements in the cephalometric analysis of sagittal deficiency. The repeatability and reproducibility of the results of the double measurement of the ANB angle were assessed on 89 cephalograms (Table 3).

The smallest variability in the assessment of repeatability, i.e., the least dependence on the physician’s error, is demonstrated in the measurement of the ANB angle (4.3%), followed by Yen (14.1%), and the largest is seen for the Tau angle (15.8%). The smallest variability in reproducibility is demonstrated by the Yen angle (0.2%), followed by ANB (0.7%), and the largest is seen for the Tau angle (1.4%), seven times greater than that for the Yen angle. At the same time, the highest accuracy is characteristic of ANB (5%), followed by Yen (14.3), and the lowest is achieved by Tau (17.2%) considering the overall variability in individual parameters. Assuming the general guidelines for the quality of measurement systems (correct below 10%, satisfactory 10–30%, requiring improvement above 30%), it should be considered that only the summary assessment of the ANB angle (6.4%) is acceptable. The results obtained for the Yen angle (30.2%) and the Tau angle (33.7%) require improvement and do not qualify these measurements for diagnostic use.

The evaluation of the R&R parameter indicates two perceptible levels of measurement error. The diversity of patients comes to the fore. The diverse skeletal configuration has the greatest impact on the reliability of determining anthropometric points and the angular measurement values obtained using them. The second factor influencing the level of reliability error (repeatability and reproducibility) after the analysis of the R&R index should be considered the error due to the doctor’s skills. A precise evaluation of the index allows us to determine which orthodontists from the entire group made the greatest error (Table 2, line 4). R&R allows for the identification of sources of error and the improvement of standards of cephalometric analysis.

### 4.3. Reproducibility Analysis

The following statistical tools were used to assess the reproducibility of using the angular measurements ANB, Tau and Yen in assessing sagittal discrepancies: R2, ICC.

In the case of the determination coefficient R2 (Table 3), values were as follows: Yen, ANB and Tau (962, 916, 907). The R squared (R^2^) statistical test, also known as the coefficient of determination, was used to assess reproducibility. It assesses the strength of the relationship between the results of the first and second measurements of the same parameter under the same conditions.

The R^2^ value ranges from 0 to 1. A value close to 1 means that the model explains the variability of the data well, while a value close to 0 suggests that the model does not explain the variability in the data.

When interpreting the obtained results (Table 4), R^2^ informs us what percentage of the variability of the dependent variable is explained by the independent variables. The data obtained for all angles indicate that the adopted model for assessing sagittal inconsistency using the ANB, Tau and Yen angles fits the data well. This indicates excellent reproducibility.

A limitation of the R^2^ indicator is the lack of information on the causality of differences in the reproducibility of measurements. It only informs us about the relationship between individual series of measurements and different assessors.

In order to examine the reproducibility of the determination of the ANB, Tau and Yen angular measurements, the ICC (intraclass correlation coefficient) was used.

In the context of cephalometric measurements, the ICC evaluates how consistent the measurements made by different examiners or under different conditions are.

ICC can be calculated in different forms, depending on the context and structure of the data:

ICC (1): Used when measurements are made by different examiners and each examiner evaluates different units.

ICC (2): Used when measurements are made by different examiners but all evaluations refer to the same units (e.g., cephalometric measurements in the same patients).

ICC (3): Used when measurements are made by the same examiners and all evaluations refer to the same units.

When interpreting the obtained results, the ICC parameter values can be determined in the range from 0 to 1:

The individual ranges of the obtained values indicate the following:

0.0–0.20: Poor agreement.

0.21–0.40: Moderate agreement.

0.41–0.60: Good agreement.

0.61–0.80: Very good agreement.

0.81–1.00: Excellent agreement.

Regarding the reproducibility of the measurements, the results of statistical analysis using ICC (Table 5) established a decline in the following order: ANB, Yen and Tau (0.946; 0.721; 0.689).

The obtained results allow us to state that excellent reproducibility can be achieved when determining sagittal discrepancy by assessing the ANB angle (0.946). The remaining measurements of the Tau angle (0.689) and Yen angle (0.721) are characterized by lower measurement reproducibility, which results in lower reliability in the assessment of sagittal relations of the maxillary bases.

### 4.4. Sensitivity and Specificity Analysis

The diagnostic utility of the entire classification system, consisting of the three tests, was determined using the following coefficients:

- Cumulative sensitivity CSens;
- Cumulative specificity CSpec.

Cumulative sensitivity is the fraction of cases that were correctly assigned to a given diagnostic group, and cumulative specificity is the fraction of cases that were correctly rejected as not belonging to a given diagnostic group.

The cumulative sensitivity and specificity for a classification system that predicts membership in one of the three groups based on the Tau angle value were calculated by taking the specificity values for each of these groups and averaging them. Because the individual groups had different numbers of observations, a weighted average was used, where the weight (wi) was the number of observations in each group. The diagnostic utility of the Tau and Yen angle classification system for assigning patients to one of three skeletal classes was determined using cumulative sensitivity and specificity coefficients. Sensitivity is the fraction of cases that were correctly assigned to a given diagnostic group, and specificity is the fraction of cases that were correctly excluded as not belonging to a given diagnostic group. The ANB angle showed the highest sensitivity (1), followed by Yen (0.709) and Tau (0.702). Similarly, the highest specificity was shown by ANB (1.0), followed by Yen (0.824) and Tau (0.819) (Table 6).

Cumulative sensitivity and specificity of the patient occlusion classification system were calculated based on the Tau angle value as follows:

$${Cum}_{Spec}=\frac{\sum_{k=1}^{n}w_{k}\cdot {Spec.}_{i}}{\sum_{k=1}^{n}w_{k}}=\frac{847\cdot 0.361+1629\cdot 0.825+1439\cdot 0.764}{847+1629+1439}=0.702,\;{Cum}_{Sens}=\frac{\sum_{k=1}^{n}w_{k}\cdot {Sens.}_{i}}{\sum_{k=1}^{n}w_{k}}=\frac{847\cdot 0.855+1629\cdot 0.791+1439\cdot 0.829}{847+1629+1439}=0.819$$

However, it should be remembered that the evaluation of the classification system translates into clinical decisions. Sometimes, more important than the statistics themselves is how the system affects the treatment of patients.

Tau angles of 30° to 33°, >33° and <30° indicate skeletal malocclusions of class I, II and III, respectively.

Both the Tau angle (Table 7) and the Yen angle (Table 8) changed the classification of individual skeletal classes in relation to that determined by the ANB angle. These changes were statistically insignificant.

Cumulative sensitivity and specificity of the patient occlusion classification system based on the Yen angle value:

$${Cum}_{Spec}=\frac{\sum_{k=1}^{n}w_{k}\cdot {Spec.}_{i}}{\sum_{k=1}^{n}w_{k}}=\frac{893\cdot 0.375+1412\cdot 0.776+1611\cdot 0.834}{893+1412+1611}=0.709,\;{Cum}_{Sens}=\frac{\sum_{k=1}^{n}w_{k}\cdot {Sens.}_{i}}{\sum_{k=1}^{n}w_{k}}=\frac{893\cdot 0.845+1412\cdot 0.841+1611\cdot 0.798}{893+1412+1611}=0.824$$

Yen angles of 121° to 126°, <121° and >126° indicate skeletal malocclusions of class I, II and III, respectively.

Determining the appropriate sensitivity and specificity of the measurements has significant implications for clinical evaluation.

The chance of correctly classifying a patient with a Yen angle > 126° to class III (mandibular prognathism) is twenty-four times greater compared to that of classifying a patient with a Yen angle ≤ 126° (OR = 24.0). The chance of correctly classifying a patient with a Yen angle < 121° to class II (mandibular retrognathism) is nineteen and a half times greater compared to that of classifying a patient with a Yen angle ≥ 121° (OR = 19.6). The results of classifying patients into three skeletal classes based on the value of the Yen angle are presented in Table 8.

The Yen angle had the highest sensitivity for correctly classifying patients to class III compared to that of the ANB angle. The sensitivity of the Yen angle to classify the patient to class III based in the ANB angle was lower than that of Tau. The Tau angle had the highest sensitivity for classifying patients into class II compared to the ANB angle, and the Yen angle had a lower sensitivity. The Tau angle had the highest specificity in relation to ANB for determining class I, and the Yen angle for determining class II.

In order to obtain cumulative results of repeatability and reproducibility and to avoid the problem of multiple comparisons leading to a decrease in the nominal significance level of each of the set of related tests in direct proportion to their total number in subsequent studies, one can apply the Bonferroni correction.

This method is resistant to interdependencies between test results, at the cost of reduced power of the tests.

The method comes down to dividing the nominal significance level α of each of the related tests by the total number of tests m.

## 5. Discussion

The assessment of sagittal discrepancy plays a key role in orthodontic assessment and treatment planning [1,2,22,23,24,25,26,27,28,29,30,31,32,33,34,35]. The conclusions of a systematic review conducted in 2022 by Kotuła et al. [1] indicate the need for comparative analyses of the cephalometric measurements discussed in this study with those exhibiting a high evidential value in past studies. The review authors indicate the need for randomized controlled trials or prospective clinical trials in comparable groups of patients comparing the diagnostic values of individual measurements. They also indicate that the studies should be performed by experienced and newly trained orthodontists. Studies should assess significant differences in the analyses performed both by each investigator and between physicians. The need to use standardized methodological criteria and compare the significance of individual measurements using the same statistical analysis was also considered important1. Due to the errors in determining cephalometric points that affect the reliability of angular measurements assessing sagittal incongruence of maxillary bases, less error-sensitive points and measurements in cephalometric analysis are systematically sought. In this study, the recommendations resulting from the suggestions of the review authors were followed1. Tau and Yen angular measurements based on the new cephalometric points G and M included in the study in relation to the ANB angle were selected based on their easier plotting compared to the SAR and W angles. Previous studies have indicated a lower susceptibility to changes related to growth or orthodontic treatment of the anatomical points that form them [1,2,22,23]. However, in this study, the possibility of error related to the belonging of the patients to a Caucasian ethnic group could not be avoided. Therefore, the obtained results should not be generalized. The differences in the results presented in this paper compared to previous studies result from the size of the patient groups and the average age of the patients, generalizations related to the lack of gender determination, the inability to perform a comparative analysis of photos of the same patient and the number of orthodontists participating in the study. The differences also result from the generally accepted research methodology. In previous publications [2,22,23,32], the repeatability and reproducibility of individual anthropometric points were assessed, which indicated the reliability of angular measurements. The current study focused on the assessment of angular parameters themselves.

Studies conducted by Maheen Ahmed et al. [36] on various cephalometric analyses consistently showed that the ANB angle is the most accurate and reliable indicator of maxillomandibular relationships in the sagittal plane. Similar results were obtained by Kotuła et al. [2] comparing the reliability of the ANB and Tau angles. The results of our study also support this conclusion. The ANB angle measurements showed smaller errors compared to the Tau and Yen angle measurements, mainly due to a narrower coaxial dispersion of angle measurements determined by the same and different observers. The results of the ANB, Tau and Yen angle measurements based on the R&R analysis, including their repeatability (4.3%; 15.8%; 14.1%), reproducibility (0.7%; 1.4%; 0.2%), inter-patient variability (93.6%; 66.5%; 69.8%) and total reproducibility and repeatability (6.4%; 33.7%; 30.2%), indicate a direct dependence of all angles on individual variability. Kotuła et al. [2] reached similar conclusions when publishing the results of ANB and Tau measurements, including repeatability (1.61%; 4.3%), reproducibility (0.92%, 3.94%), inter-patient variability (97.47%; 91.76%) and total reproducibility and repeatability (2.53%; 8.24%), emphasizing the dependence of the ANB and Tau angles on individual variability. Both studies emphasized the greater effectiveness of the ANB angle as a standard parameter for assessing sagittal relationships. The reliability of the data was further increased by a double-blind study design, in which observers did not know either the clinical parameters of the patients or the purpose of the analyses, which allowed them to perform an objective assessment despite several limitations that influenced the final assessment value. The study by Kotuła et al. [2] analyzed the repeatability and reproducibility of the sagittal discrepancy parameters of the ANB and Tau angles based on the Dahlberg formula (0.265–0.665; 0.891–1.639) and ICC (0.841–1; 0.147–0.624). The authors found that the orthodontists participating in the study demonstrated significantly higher accuracy in measuring the ANB angle compared to the Tau angle. All the above-mentioned indicators indicated a three-fold higher reliability of measuring the ANB angle compared to the Tau angle. They considered that this discrepancy is influenced by the smaller variability in the horizontal coordinates of points, which have the greatest impact on the angle measurement error. In the current study, when assessing the reliability of measurements, the assessment of the position of points in the Cartesian system was not taken into account. The obtained values for the ANB and Tau angles based on the Dahlberg formula (0.68%;2.63%) and the ICC (0.946; 0.689) confirm the previous conclusions.

The intention of searching for cephalometric measurements based on new anatomical points is to minimize the errors of ANB measurements related to changes in the position of points A, B and N, especially for patients in the growth period [1,2,22,23,33]. Gupta P. et al. [23]. Neela et al. [22] and Kumar et al. [32] suggest higher repeatability in determining points M and G, located near the central positions in the maxilla and mandibular symphysis. Gupta et al. [23] argue for the better reliability of the T point, which does not change its position after 4–5 years of age, and thus suggest that the angle formed using this point should be characterized by the highest reliability. Such a hypothesis, especially when considering the reservations regarding ANB, requires scientific confirmation. The results obtained by Kotuła et al. [2] in an attempt to verify it did not prove the greater value of the Tau angle in the assessment of sagittal inconsistency in the maxillary bases. Also in the current study, we encountered difficulties, among others, in correctly locating the T point, which could have directly influenced the measurement values and lowered the diagnostic value of this parameter. Comparisons of the repeatability of ANB, Tau and Yen using the R&R assessment (4.3%, 15.8%, 14.1%) and, analogously, reproducibility (0.7%; 1.4%; 0.2%) indicate the lower reliability of the assessment of the relationship of the maxillary bases with the sagittal plane using the new parameters. Moreover, the study by Kotuła et al. [2] indicated that there is no clear evidence to support the claim that the Tau angle is resistant to mandibular rotation or consistently gives stable results for the correct determination of sagittal defects. Both the previous [2] and the current studies emphasize that errors related to the identification of anthropometric landmarks are more significant than errors in the repeatability or reproducibility of landmarks in the assessment of ANB and Tau angles. Gupta [23] et al. and Kotuła et al. [2] attempted to assign the appropriate Tau angle values to one of three skeletal classes. The current study, conducted on a selected group of patients with orthognathic faces without taking into account the assessment of the vertical component and its influence on sagittal relations based on ANB angle measurements, slightly changed the marginal values for skeletal classes determined using the Tau and Yen angles (Table 1).

The R2 coefficient assessment allowed us to accept the results of previous studies by Neel [22] and Kumar [33], establishing its value at 0.962, which indicated a higher value compared to those of ANB (0.916) and Tae (0.907). However, in the analysis (R&R) of Yen and Tau angles, the repeatability and reproducibility values and the doctor–patient relationship were almost five times lower in determining the accuracy compared to those of the ANB angle. The value of this study is the greater reliability of the results associated with a double-blind study, in which the observers were not aware of the clinical parameters of the patients or the purpose of the analyses, which ensured an unbiased assessment.

A further value of this study is the assessment of the sensitivity and specificity of individual measurements. By analyzing the cumulative values of sensitivity and specificity, we can evaluate the classification systems of malocclusions using Tau and Yen angles in relation to the classification based on ANB. Thanks to this, we are able to assess the ability of Tau and Yen measurements to correctly identify cases (sensitivity) and to correctly exclude cases that should not be classified as malocclusions (specificity). Sensitivity values for determining class II or III malocclusions using the Tau/Yen index (0.825; 0.764/0.776; 0.834) indicate that the correct classification of patients into the appropriate class was performed for 82.5%, 76.4%/77.6% and 83.4% of cases, respectively. The higher the sensitivity of the measurement, the lower the risk of omitting people with skeletal class II or III during the diagnosis. Such high values indicate that both parameters effectively identify malocclusions, although a significant proportion of patients are incorrectly assessed. The causes of this phenomenon can be seen in the so-called borderline cases between individual malocclusions. We can also expect a similar assessment when assessing the previous ANB angle measurement. High sensitivity means that the risk of overlooking the actual malocclusion is low, which is beneficial for clinical use in orthodontic diagnostics. In assessing the sensitivity of the Tau measurement, Kumar 32 indicated a sensitivity of 100%, which was not confirmed in the current study. By assessing the specificity of the measurements, we are able to avoid false positive results, i.e., incorrect classification of healthy people as presenting malocclusions. The specificity values for determining class II or III malocclusions using the Tau/Yen index (0.791; 0.829/0.850; 0.798) indicate that each of the parameters correctly identifies 79.1%, 82.9%/85.0% and 79.8% people who do not have these specific malocclusions in relation to the sagittal plane. At the same time, from 15% to almost 21% of healthy people may be misdiagnosed as requiring orthodontic intervention due to sagittal incongruence. In assessing the specificity of the Tau measurement, Kumar [32] reported a specificity of 98%, which was not confirmed in the current study.

The clinical application of the obtained results of sensitivity and specificity for both parameters may be associated with increased diagnostics using other previously used angular or linear measurements, including ANB or Wits analysis. This will be particularly important in the diagnosis of borderline values between class I and II or I and III.

Both new parameters are perfectly suited for use in screening tests, minimizing the risk of omitting patients with sagittal relation malocclusions.

### 5.1. Limitations

The main limitations of this study were the number of patients, although obtaining almost 4000 measurements for each variable allows for some generalizations. Another limitation is the different age of the patients, which prevents unambiguous generalization of the obtained results, at least to the group of children in puberty. The inability to compare photos of the same patient taken before and after the growth period constitutes an additional limitation in the assessment of changes in the position and displacement of individual cephalometric points with age. Typically, changes in the positions of points A in relation to the *X*-axis in the longitudinal dimension and N in relation to the *Y*-axis in the vertical dimension are observed in connection with changes in bone layering in these anatomical areas related to growth. Single examinations without repetition after the growth period constitute a limitation for the potential possibility of identifying changes in the positions of points M and G (especially in relation to the vertical component of the *Y*-axis) during the growth period. The fact that the examinations were conducted only by 22 orthodontists and the limited intensity of training can also be considered limitations. This is particularly important when comparing the commonly known ANB angle and introducing new points M, G and T. It may also have an impact on the interpretation of the obtained results. The limitations of this study include the restriction of the analyses to the repeatability and reproducibility of cephalometric measurements without taking into account the reliability of entering cephalometric points in relation to the x- and y-axes.

Limitations can also be seen in the cephalometric analysis program itself, although its reliability did not raise concerns for the authors. Therefore, further research is warranted, in particular randomized controlled trials (RCTs) comparing alternative methods of assessing sagittal discrepancies in sufficiently large groups. The accuracy of the determination of the T point was also a limitation due to the low precision of radiographs in the studied area.

### 5.2. Future Research Directions

Further research is warranted, especially randomized controlled trials (RCTs) comparing alternative methods of assessing sagittal discrepancies. They should include an increased sample size, both in terms of the number of orthodontists participating in the study and the number of patient cases, taking into account the relationship between sagittal discrepancy and vertical discrepancy. The results obtained in individual groups could be improved regarding generalizability and statistical power. Studies should be conducted with blinding of the radiograph, the patient and the assessing orthodontist. They should also include the number of radiographs excluded and the reasons for excluding radiographs from assessment or for excluding analyses due to significant discrepancies from other analyses. The introduction of standardized measurement training for orthodontists with a duration of more than the 5 h offered in the current study should be considered crucial in designing future studies. This should reduce interobserver variability and improve the consistency and reliability of the results. Future studies should consider the possibility of comparing the use of digital tools for cephalometric analysis with manual measurements, which can increase accuracy and reduce the number of human errors. The possibility of using AI to conduct cephalometric analyses should also be considered. The authors also see the need to conduct complementary studies to assess the long-term reliability of these angles in predicting the results of orthodontic treatment, which would provide valuable clinical information for practicing orthodontists. Further studies should consider the possibility of integrating additional angles currently used in the assessment of sagittal discrepancies. Their inclusion may enable a more comprehensive assessment of sagittal discrepancies and improve the accuracy of the classification while helping to indicate the right measurements that should be used in selected clinical situations, supporting the possibility of proper planning for orthodontic treatment.

## 6. Conclusions

The presented study confirms the possibility of using the new Tau and Yen cephalometric measurements in the assessment of sagittal discrepancy as a supplement to the previously existing ANB angle measurement.

In assessing the reliability of individual angular measurements, the ability to position anthropometric points based on knowledge and experience of orthodontists comes to the fore, as doctors find it easier to use existing markers. In order to improve the reliability of measurements, the training of doctors in the positioning of landmarks should be carried out, and computer programs should be prepared to support doctors in visualizing the entered points. With the development of AI, the training of artificial intelligence in this area can also be started.

The average difference in the results of the assessment of the repeatability of the results of all the analyses discussed is at the level of 0.03 to 011 mm, which allows for the practical use of each of these parameters for the assessment of sagittal discrepancy; however, the new measurements are characterized by a larger error range, which in extreme cases prevents their use in orthodontic diagnostics. Due to the limitations of this study, the assessment of individual parameters should be approached critically. Special care should be taken with patients of developmental age and in the analysis of sagittal discrepancy with the height of the maxillary bases considering the vertical movement of the G point with growth.

High sensitivity at the level of 80% and specificity at the level of 70% for Tau and Yen measurements allows for the effective identification of patients with malocclusions due to sagittal discrepancy. The assessment system shows a better ability to detect defects than to exclude them; therefore, in extreme situations, it should be compared with the current standards. Clinically, it has a higher application in the diagnosis of class II and III defects.

The results of the ANB angle measurement show lower error rates both in repeatability and reproducibility compared to the results for the Yen and Tau angles.

## Figures and Tables

**Table 1:** Criteria for dividing patients into skeletal groups, numbers (N) and fractions (%) of classification results for 89 patients based on duplicate ANB angle measurements performed by 22 physicians and the result of the chi-squared test of the observed distribution with a uniform distribution.

Angle (°)	Class I Normocclusion	Class II Mandibular Retrognathia	Class III Mandibular Prognathism	*p*
ANB	0° ≤ i ≤ 4°	>4°	<0°	0.957
	N = 1303 (33.3%)	N = 1296 (33.1%)	N = 1317 (33.6%)	

**Table 2:** Criteria for dividing patients into groups.

Angle (°)	Class 1	Class 2	Class 3
ANB	≥0	>4	<0
	≤4	≤8	≥−4
Yen	≥121	<121	>126
	≤126	≥117	≤129
Tau	≥30	>33	<30
	≤33	≤36	≥27

**Table 3:** Results of the analysis (R&R) of the repeatability and reproducibility of the double measurement of the ANB, Tau and Yen angles performed by 22 physicians on 89 cephalograms.

	ANB	Yen	Tau
Reproducibility (the same dentist	4.3%	14.1%	15.8%
Repeatability (dentists)	0.7%	0.2%	1.4%
Objects (patients)	93.6%	69.8%	66.5%
Interaction (dentists/patients)	1.4%	15.8%	16.5%
R&R	6.4%	30.2%	33.7%

**Table 4:** Comparison of the reproducibility of the angle measurements of ANB, Yen and Tau in two subsequent analyses performed by 22 orthodontists in a group of 89 patients and the value of the coefficient of determination R2.

	ANB	Yen	Tau
R^2^	0.916	0.962	0.907
Percentage of repetition in the second study	91.6%	96.2%	90.7%

**Table 5:** ICC for individual angles ANB, Yen and Tau.

	ANB	Yen	Tau
ICC	0.946	0.721	0.689

**Table 6:** Cumulative values of sensitivity and specificity of ANB, Tau and Yen angles.

	ANB	Tau	Yen
Cumulative sensitivity	1	0.702	0.709
Cumulative specificity	1	0.819	0.824

**Table 7:** Number (fraction) of respondents in groups differing in skeletal class assessment based on ANB angle and Tau angle values, chi-squared test of independence results and odds ratio values and their 95% confidence intervals.

	Class I 0° ≤ ANB ≤ 4° N = 1303	Class II and III ANB < 0° or ANB > 4° N = 2613	*p*-Value	OR [95% CI]
30° < Tau < 33°	470 (36.1%)	378 (14.5%)	<0.001	3.34 [2.85; 3.90]
Tau < 30° or Tau > 33°	833 (63.9%)	2235 (85.5%)		1.00 (ref.)
Sensitivity *Sens* = 0.361, specificity *Spec* = 0.855				
	**Class II**	**Class I and III**	*p*-value	OR [95% CI]
	**ANB >4°**	**ANB ≤ 4°**		
	N = 1317	N = 2599		
Tau > 33°	1086 (82.5%)	543 (20.9%)	<0.001	17.08 [15.01; 21.11]
Tau ≤ 33°	231 (17.5%)	2056 (79.1)		1.00 (ref.)
Sensitivity *Sens* = 0.825, specificity *Spec* = 0.791				
	**Class III**	**Class I and II**	***p*** **-value**	**OR [95% CI]**
	**ANB < 0°**	**ANB ≥ 0°**		
	N = 1296	N = 2620		
Tau < 30°	990 (76.4%)	449 (17.1%)	<0.001	15.64 [13.28; 18.42]
Tau ≥ 30°	305 (23.6%)	2171 (82.9%)		1.00 (ref.)
*Sensitivity Sens* = 0.764, specificity *Spec* = 0.829				

**Table 8:** Number (fraction) of respondents in groups differing in skeletal class assessment based on ANB angle and Yen angle values, chi-squared test of independence results and odds ratio values and their 95% confidence intervals.

	Class I 0° ≤ ANB ≤ 4° N = 1303	Class II and III ANB < 0° or ANB > 4° N = 2613	*p*-Value	OR [95% CI]
121° ≤ Yen ≤ 126°	489 (37.5%)	404 (15.5%)	<0.001	3.28 [2.82; 3.83]
Yen < 121° or Yen > 125°	814 (62.5%)	2209 (84.5%)		1.00 (ref.)
Sensitivity *Sens* = 0.375, specificity *Spec* = 0.845				
	**Class II**	**Class I and III**	***p*** **-value**	**OR [95% CI]**
	**ANB > 4°**	**ANB ≤ 4°**		
	N = 1317	N = 2599		
Yen < 121°	1022 (77.6%)	390 (15.0%)	<0.001	19.62 [16.58; 23.22]
Yen ≥ 121°	295 (22.4%)	2209 (85.0%)		1.00 (ref.)
Sensitivity *Sens* = 0.776, specificity *Spec* = 0.850				
	**Class III**	**Class I and II**	***p*** **-value**	**OR [95% CI]**
	**ANB < 0°**	**ANB ≥ 0°**		
	N = 1296	N = 2620		
Yen > 126°	1081 (83.4%)	530 (20.2%)	<0.001	19.83 [16.65; 23.61]
Yen ≤ 126°	215 (16.6%)	2090 (79.8%)		1.00 (ref.)
Sensitivity *Sens* = 0.834, specificity *Spec* = 0.798				

## Data Availability

Data regarding this article are available from the corresponding author.
